# Improving Therapy of Pharmacoresistant Epilepsies: The Role of Fenfluramine

**DOI:** 10.3389/fphar.2022.832929

**Published:** 2022-05-20

**Authors:** Gianluca Dini, Eleonora Tulli, Giovanni Battista Dell’Isola, Elisabetta Mencaroni, Giuseppe Di Cara, Pasquale Striano, Alberto Verrotti

**Affiliations:** ^1^ Department of Pediatrics, University of Perugia, Genoa, Italy; ^2^ Pediatric Neurology and Muscular Diseases Unit, IRCCS “G. Gaslini” Institute, Genoa, Italy; ^3^ Department of Neurosciences, Rehabilitation, Ophthalmology, Genetics, Maternal and Child Health, University of Genoa, Genoa, Italy

**Keywords:** fenfluramine, pharmacoresistant epilepsy, Dravet syndrome, Lennox-Gastaut, anti-seizure medication (ASM)

## Abstract

Epilepsy is among the most common neurological chronic disorders, with a prevalence of 0.5–1%. Despite the introduction of new antiepileptic drugs during recent years, about one third of the epileptic population remain drug-resistant. Hence, especially in the pediatric population limited by different pharmacokinetics and pharmacodynamics and by ethical and regulatory issues it is needed to identify new therapeutic resources. New molecules initially used with other therapeutic indications, such as fenfluramine, are being considered for the treatment of pharmacoresistant epilepsies, including Dravet Syndrome (DS) and Lennox-Gastaut Syndrome (LGS). Drug-refractory seizures are a hallmark of both these conditions and their treatment remains a major challenge. Fenfluramine is an amphetamine derivative that was previously approved as a weight loss drug and later withdrawn when major cardiac adverse events were reported. However, a new role of fenfluramine has emerged in recent years. Indeed, fenfluramine has proved to be a promising antiepileptic drug with a favorable risk–benefit profile for the treatment of DS, LGS and possibly other drug-resistant epileptic syndromes. The mechanism by which fenfluramine provide an antiepileptic action is not fully understood but it seems to go beyond its pro-serotoninergic activity. This review aims to provide a comprehensive analysis of the literature, including ongoing trials, regarding the efficacy and safety of fenfluramine as adjunctive treatment of pharmacoresistant epilepsies.

## Introduction

Epilepsy is one of the most common chronic neurological disorder, with a significant socioeconomic and psychological impact worldwide. It affects over 70 million people, one-third of which is drug-resistant (Löscher et al., 2020; Fattorusso et al., 2021). The definition for drug resistant epilepsy (DRE) is not unique, but it is defined by the International League Against Epilepsy (ILAE) as failure of adequate trials of two tolerated, appropriately chosen and used antiepileptic drug schedules (whether as monotherapies or in combination) to achieve sustained seizure freedom (Kwan et al., 2010). DRE reduces quality of life and it is a potential life-threatening condition. Nowadays, despite the advances in the field of epilepsy and the recent approval of new antiseizure medications (ASMs), DRE still represents a major problem (Yoo and Panov 2019; Fattorusso et al., 2021). The exact incidence and prevalence of DRE are uncertain, due to the non-univocal definitions and misdiagnosis (Dalic and Cook 2016). In a recent epidemiological systematic review by Kaliani et al. the pooled prevalence of DRE among epileptic patients was 30%. The pooled incidence proportion was 15% in children and 34% in adults, with an overall pooled incidence of 20% (Kalilani et al., 2018). These results were consistent with those frequently reported in the literature. Several risk factors for DRE have been identified, for example age at epilepsy onset (<1 year) or epilepsy aetiology. Patients with symptomatic epilepsy had 3 times-increased risk for DRE compared with patients with idiopathic epilepsy (Chen et al., 2018; Kalilani et al., 2018). Not surprisingly Lennox-Gastaut syndrome (LGS), Dravet Syndrome (DS), early infantile epileptic encephalopathy or Rasmussen encephalitis are almost pharmacoresistant (Dalic and Cook 2016). Also the coexistence of neuropsychiatric disorders such as intellectual disability or Attention Deficit Hyperactivity Disorder (ADHD) is related to the risk of DRE (Matricardi et al., 2020). Other risk factors include a history of febrile seizure, status epilepticus, abnormal EEG, abnormal neuroimaging test results (Kalilani et al., 2018) or an inadequate response to the initial ASM therapy and time to achieving seizure freedom (Kwan and Brodie 2000; Schmidt 2007). Sex and seizure type were not associated with risk of DRE, although focal seizures were suggested to have a higher risk than generalized seizures (Kalilani et al., 2018). Family history of epilepsy is a controversial risk factor of DRE (Fattorusso et al., 2021). The heterogeneity of seizure types and epileptic syndromes, the presence of comorbidities, the multifactorial genesis and the difficulty to understand its exact causal mechanism make DRE management and treatment extremely challenging. This is particularly true in pediatric patients. Indeed, epileptic syndromes like LGS or DS, that are often associated with pharmacoresistant epilepsy, occur in pediatric age. Moreover, ASMs are often used in an off-label manner in children due to the lack of clinical trials in this population. Treatment options available for DRE patients are polytherapy, surgical therapy or alternative therapy, as vagus nerve stimulation or ketogenic diet (López González et al., 2015; Löscher et al., 2020; Verrotti et al., 2020; Fattorusso et al., 2021). Polytherapy should be considered as first line of treatment. When choosing the most appropriate ASMs combination several factors should be kept in consideration, as efficacy, mechanism of action, pharmacokinetics, potential synergic interaction (for example Valproate plus Lamotrigine) and the risk of an additive adverse event profile. The addition of a fourth drug should be avoided (López González et al., 2015; Park et al., 2019; Verrotti et al., 2020). Recently new molecules have been approved as ASMs either as add-on therapy or initial monotherapy. New ASMs have been studied in several randomized controlled trials (RCT) and, compared with conventional ASMs, seem to have a better pharmacological profile: linear pharmacokinetics, less drug-drug interactions, different mechanisms of action and better tolerability profiles, which are important advantages for polytherapy (Park et al., 2019). Perampanel has been recently approved as add-on treatment in patients with focal seizures (with or without secondarily generalization) and primary generalized tonic-clonic seizures. It is well tolerated and it has been proved to be effective on idiopathic generalized and focal DRE (French et al., 2015; Krauss et al., 2018; Operto et al., 2020). Brivaracetam has been approved as adjunctive treatment in adults and pediatric patients aged 4 years and older with focal onset seizures. It seems to show a positive response also in patients affected by some encephalopathic epilepsies (Tulli et al., 2021; Verrotti et al., 2021). Another emergent promising ASM is Cannabidiol (CBD). Several trials have proved its effectiveness in DS and LGS patients (Devinsky et al., 2017; Lattanzi et al., 2019; Verrotti and Striano 2021). A highly purified plant-based form of oral CBD formulation was approved by the Food and Drug Administration (FDA) in 2018 and the European Medicines Agency (EMA) in 2019 for the treatment of seizures associated with DS and LGS (Contin et al., 2021). Cenobamate, a novel tetrazole-derived carbamate compound, has been recently approved in the United States for the treatment of partial-onset seizures in adult patients [Keam 2020] (Löscher et al., 2020). Fenfluramine (FFA), first used as an antidepressant and later as an appetite suppressant, was withdrawn from the market because of cardiac side effects. Nowadays, FFA is reintroduced as ASM at a lower dosage (Odi et al., 2021). The use of new ASMs in the pediatric population is often limited by different pharmacokinetics and pharmacodynamics and by ethical and regulatory issues. The aim of this review is to provide a comprehensive analysis of the current literature regarding the FFA pharmacologic profile and the clinical data regarding its safety and efficacy which may justify its use as an ASM, especially in pediatric population.

### Literature Search

Electronic databases MEDLINE, EMBASE, and the Clinical Trial Database were systematically searched to identify relevant studies published through November 2021. Papers were searched using the following terms: “fenfuramine”, “pharmacodynamics and fenfuramine”, “pharmacokinetics and fenfuramine”, “Dravet syndrome”, “Lennox Gastaut syndrome”. The abstracts of retrieved references were reviewed and prioritized by relevant content and by the quality of evidence reported. Reference lists of the selected articles were used to search for further relevant papers. Only articles in English were reviewed. Additional information was also obtained from the websites of US and European Union agencies (US Food and Drug Administration and European Medicines Agency).

### Fenfluramine: Pharmacodynamics and Pharmacokinetics

**FIGURE 1:**
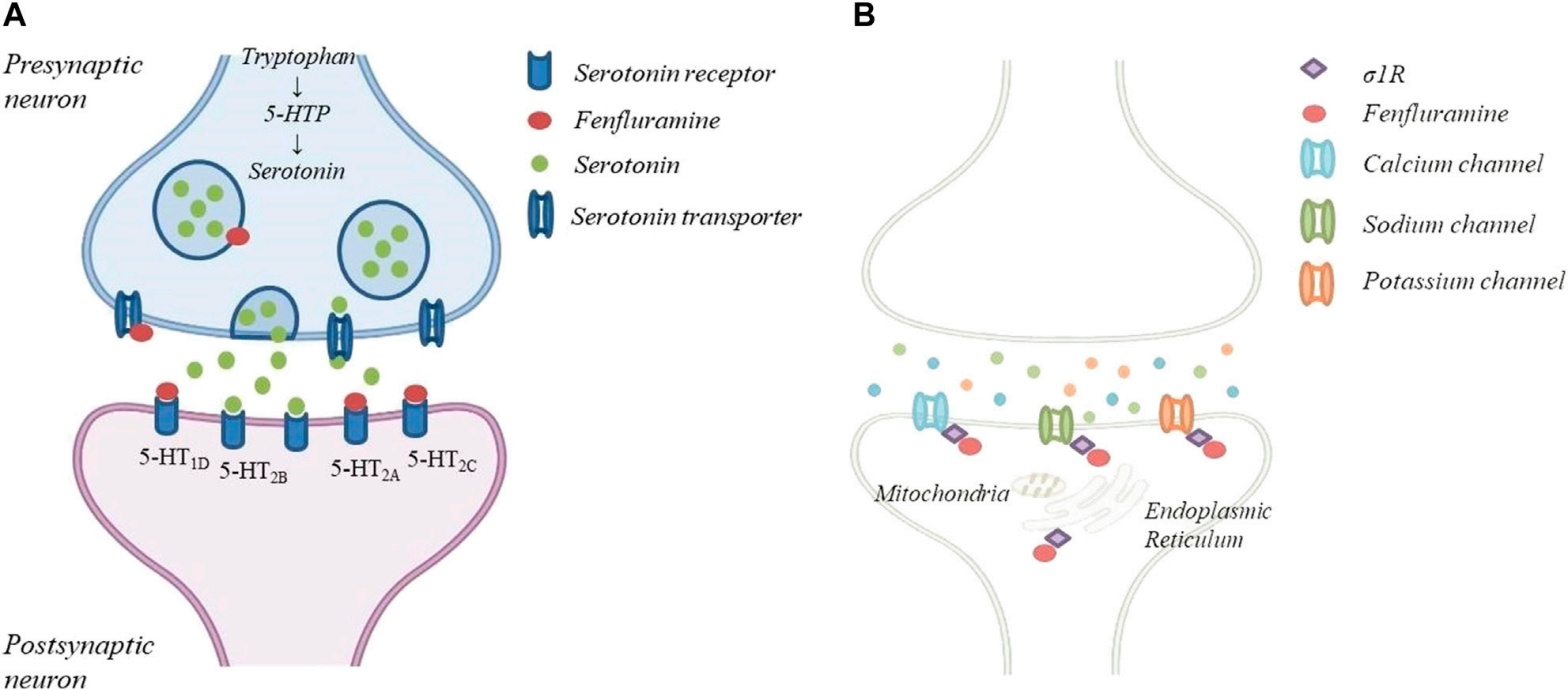
The mechanism of action of FFA: **(A)**FFA causes the release of serotonin by disrupting vescicular storage, reversing serotonin transpoter function and a agonist of specific serotonin receptor; **(B)**FFA can regulate the activity od 1R located in the mitochondria-associated endoplasmic reticulum and several ion channels.

FFA is a derivative of amphetamine and its chemical name is 3-trifluoromethyl-N-ethylamphetamine. It is a racemic mixture of dexfenfluramine and levofenfluramine (Odi et al., 2021; Balagura et al., 2020). The D-enantiomer dexfenfluramine promotes serotonin-mediated neurotransmission by inhibiting serotonin (5-HT) reuptake and it has been used as an appetite suppressant to treat obesity (Garattini et al., 1987). The L-enantiomer, which lacks serotoninergic activity, can suppress dopaminergic transmission (Invernizzi et al., 1989; Wurtman and Wurtman 2018). The racemic mixture, now proposed as ASM, acts on serotonin receptors (5HT2R) and on sigma 1 receptors (σ1R), as demonstrated *in vitro* and *in vivo* models of DS (Sourbron et al., 2017; Rodríguez-Muñoz et al., 2018; Martin et al., 2020) (Figure 1). In particular, FFA and its metabolite norfenfluramine exert the antiseizure activity as agonist of 5-HT1D and 5-HT2C type receptors, while the 5-HT2B receptor seems not to be involved. The role of the 5-HT2A receptor is not fully understood (Sourbron et al., 2017). In addition, FFA and norfenfluramine can regulate the activity of σ1R, a class of receptor that exert a modulatory effect on neurotransmitters involved in the genesis of seizures. In a mouse model of induced seizures, FFA seems to disrupte the association of the σ1R with NR1 subunits of glutamate N-methyl-D-aspartate receptors (NMDAR), restricting NMDAR activity. Thanks to this mechanism of action FFA seems to evade the negative side effects of direct NMDAR antagonists and may improve the quality of life of patients with DS and LGS (Rodríguez-Muñoz et al., 2018). The antagonism of σ1R by FFA was also confirmed by Soubron et all. in a SCN1a mutant Zebrafish model reproducing DS (Sourbron et al., 2017). On the contrary, Martin et all. demonstrated that FFA shows a positive modulation of σ1R, leading to an improvement in executive function (Balagura et al., 2020; Martin et al., 2020; Martin et al., 2021). However, the exact mechanism underlying the anticonvulsant activity of FFA is not yet completely understood (Gharedaghi et al., 2014; Rodríguez-Muñoz et al., 2018). FFA is a fat-soluble drug, it is administered orally and it is rapidly absorbed from the gastrointestinal tract. It has a good bioavailability, not affected by food intake and peak plasma concentration is observed about 3 h after a single oral dose (Gammaitoni et al., 2018; Balagura et al., 2020; Odi et al., 2021). Steady state is reached after 3–4 days of treatment (Ceulemans et al., 2012). FFA is extensively metabolized to active metabolites d-norfenfluramine and l-norfenfluramine, mostly by cytochromes CYP2D6, CYP1A2 and CYP2B6 (Boyd et al., 2019; Odi et al., 2021) and lesser by CYP2C9, CYP2C19, CYP3A4 (Balagura et al., 2020). Both FFA and norfenfenfluramine are about 50% bound to plasma proteins. The half-life of FFA is 20 h, while the half-life of norfenfluramine is longer (from 24 to 48 h), with a fast urinary excretion rate (Gammaitoni et al., 2018; Boyd et al., 2019). The fraction of the dose excreted in urine as unchanged FFA and norfenfluramine is from 6 to 24% (Balagura et al., 2020). The extensive metabolism involving different CYPs may mitigate the metabolic interactions with other ASMs. However, a moderate interaction is present when FFA, Valproate and Clobazam are used in association with stiripentol. In this case an adjustment of the FFA dosage is needed (Boyd et al., 2019). Pharmacokinetic and tolerability of FFA in children and adolescents were studied in several RCTs of patients with DS or LGS (Lagae et al., 2018; Nabbout et al., 2020). At a dose from 0.2 mg/kg/day to 0.7 mg/kg/day (with a maximum of 26 mg/day) FFA has proven to have a good pharmacological profile, with few and mild adverse events (AEs). The most common AEs were pyrexia, nasopharyngitis, decreased appetite, diarrhea, fatigue, lethargy, somnolence, and decreased weight. No valvular heart disease or pulmonary arterial hypertension were observed (Ceulemans et al., 2012; Schoonjans et al., 2017; Lagae et al., 2018; Lagae et al., 2019; Nabbout et al., 2020). Indeed, cardiac valve toxicity and pulmonary hypertension, which lead to withdrawal of FFA from the market in 1997, were achieved at higher dosages (60–120 mg/day) and they were caused by the stimulation of the 5-HT2B receptor, not involved in FFA antiseizure activity (Fitzgerald et al., 2000; Odi et al., 2021). Neverthless, a follow-up echocardiography and weigh monitoring are mandatory while treating with FFA (Balagura et al., 2020).

### Fenfluramine in Dravet Syndrome

DS is a rare developmental and epileptic encephalopathy characterized by highly treatment resistant seizures and progressive neuro-cognitive decline (Brigo et al., 2018). Children with DS have normal development in the first year of life. Seizures occur at an average age of 6 months and are usually hemiclonic or generalized tonic-clonic, triggered by fever. Over time, other seizure types appear including myoclonic, atypical absence and focal seizures. Intellectual disability and behavioural disorders also become a serious concern. DS is associated with mutations of the SCN1A gene in 70–80% of patients. SCN1A encodes the alpha1 subunit of the sodium channel and its mutation results in a broad spectrum of clinical phenotypes (Connolly 2016; Scheffer and Nabbout 2019). The increasing number of antiseizure medications in the last decades has led to the development of new successful therapies in DS including FFA (Table1). Schoonjan et al. evaluated FFA as adjunctive therapy in 9 DS patients refractory to standard AEDs. FFA yielded significant improvements, with 78% of patients having a ≥50% reduction in major motor seizure frequency for the whole duration of the treatment (Schoonjans et al., 2017). In an open-label study conducted in 4 Italian centers, FFA was added to conventional therapy in 52 DS patients, all carrying SCN1A genetic variants. In a median follow-up of 9 months 71.1%, out of 45 patients, had a ≥50% reduction in convulsive seizures, 11.1% of patients became seizure-free (Specchio et al., 2020). In a multi-centre double-blind RCT, 87 DS patients receiving a stable, stiripentol-inclusive AED regimen, were randomized to receive fenfluramine or placebo. 54% of patients treated with fenfluramine experienced a ≥50% reduction in monthly convulsive seizure frequency compared to 5% of placebo group (Nabbout et al., 2020). Efficacy and safety of FFA were assessed by Lagae et al. in 119 children and young adults with DS and seizures not completely controlled by their current regimen of AEDs. Patients were randomly assigned to receive FFA 0.2 mg/kg/day, FFA 0.7 mg/kg/day or placebo. A responder rate (≥50% seizure reduction) of 68 and 38% was reported in patients treated with FFA 0.7 mg/kg/day and FFA 0.2 mg/kg/day respectively (Lagae et al., 2019). Patients who completed any of the phase 3 core clinical trials (Lagae et al., 2019; Nabbout et al., 2020) were enrolled in an open-label extension study. A total of 232 DS patients were treated with fenfluramine at a starting dose of 0.2 mg/kg/day and subsequently increased up to a maximum of 0.7 mg/kg/day. Final results confirmed the short-term data with 64.4% of patients showing a ≥50% reduction in convulsive seizure frequency (Sullivan et al., 2020). Based on these data, the main adverse effects related to the use of FFA were decreased appetite, fatigue, diarrhea, and pyrexia. Cardiac monitoring did not reveal clinical or echocardiographic evidence of valvular heart disease or pulmonary arterial hypertension in the cohorts of patients examined.

**TABLE 1:** Main results from clinical trials for fenfuramine (FFA) use in pharmacoresistant epilepsies.

References (First Author, year)	Sample Size (age)	EE	Number of Concomitant AEDs at Baseline	Treatment Duration	Treatment Arms (Number of patients)	Global Seizure Reduction ≥50% (%)	Most Common Adverse Events
Schoonjans et al. (2017)	9 (1.2–29.8 years)	DS	2–5	Median 1.5 years	FFA 0.25–1.0 mg/kg/d (Kwan and Brodie 2000)	78%	somnolence (55.6%) anorexia (44.4%) fatigue (33.3%)
Nabbout et al. (2020)	87 (2–18 years)	DS	2–5	15 w	FFA 0.4 mg/kg/d (Scheffer and Nabbout 2019) Placebo (Specchio et al., 2020)	54% 5%	decreased appetite (44%) fatigue (26%) pyrexia (26%) diarrhea (23%)
Lagae et al. (2019) (Lagae et al., 2019)	119 (2–18 years)	DS	Mean 2.3 Mean 2.5 Mean 2.4	14 w	FFA 0.2 mg/kg/d (Schoonjans et al., 2017) FFA 0.7 mg/kg/d (Fitzgerald et al., 2000) Placebo (Fitzgerald et al., 2000)	38% 68% 12%	decreased appetite diarrhea, fatigue
Specchio et al. (2020)	45 (2.1–28.6 years)	DS	1–3	Median 9 months	FFA 0.2–0.7 mg/kg/d (Sullivan et al., 2020)	71.1%	decreased appetite (15.5%)
Sullivan et al. (2020) (Sullivan et al., 2020)	232 (2–19 years)	DS	n.a	Median 256 days	FFA 0.2–0.7 mg/kg/d^a^ (232)	64.4%	pyrexia (21.6%) nasopharyngitis (19.4%) decreased appetite (15.9%)
Lagae et al. (2018)	13 (3–17 years)	LGS	2–5	20 w (core study) 15 months (extension study)	FFA 0.2–0.8 mg/kg/d (Park et al., 2019)	62% (core study) 67% (extension study)^b^	decreased appetite (31%) decreased alertness (15%)
NCT03355209 (Knupp, 2021a)	263 (2–35 years)	LGS	1–5	14 w	FFA 0.2 mg/kg/d (89) FFA 0.7 mg/kg/d (87) Placebo (87)	28.1% 25.3% 10.3%^c^	decreased appetite, somnolence, fatigue, vomiting, diarrhea
NCT03355209 (Knupp 2021b)	170	LGS	1–7	10–12 months	FFA 0.2–0.7 mg/kg/d	51.2%^c^	Decreased appetite (16.2%) Fatigue (13.4%) Nasopharyngitis (12.6%)
Devinsky et al. (2021)	6 (2–26 years)	CDD	2–5	Mean 5.3 months	FFA 0.2–0.7 mg/kg/d	Median 90% reduction in GTCS	decreased appetite (16.6%) flatus (16.6%) lethargy (16.6%)
NCT04289467 (ClinicalTrial.gov, 2020)	Estimated 10	West syndrome	-	21 days	FFA 0.8 mg/kg/d	-	-
Geenen et al. (2021)	9 (7–24 years)	Sunflower syndrome	1–2	3 months	FFA 0.2–0.7 mg/kg/d	88.8%^d^	fatigue (40%) loss of appetite (30%) rhinorrhea (10%)

Note: a maximum of 0.4 mg/kg/d in patients receiving concomitant stiripentol; b Nine patients entered the extension study; c ≥ 50% reduction in monthly drop seizures; d Responder: ≥30% reduction in seizure activity; Epileptic Encephalopathy: EE.

### Fenfluramine in Lennox–Gastaut Syndrome

LGS is a childhood epileptic encephalopathy characterized by multiple seizure types, abnormal electroencephalographic features and cognitive impairment, leading to life-long disability (Cross et al., 2017). LGS can have different underlying etiologies, which are identifiable in 65–75% of the patients (Asadi-Pooya 2018). The most common types of seizures associated with LGS are tonic, atonic or atypical absence seizures, although other seizure types may occur. LGS is one of the most challenging epilepsy: the first-line therapy is represented by valproate, to which lamotrigine and clobazam can be added (Strzelczyk and Schubert-Bast 2021). However, prognosis remains poor and complete seizure control with resolution of neurocognitive disorders are often not achievable (Borrelli and El Tahry 2019). The promising results of fenfluramine in DS encouraged its use in LGS as well (Table1). In a phase III, multicenter, double-blind, placebo-controlled study (NCT03355209), a total of 263 patients with LGS were randomly assigned to receive FFA 0.7 mg/kg/day, FFA 0.2 mg/kg/day and placebo. FFA dosage was gradually titrated over 2 weeks and then maintained for an additional 12 weeks at a stable dosage. 25.3 and 28.1% of patients treated respectively with FFA 0.7 mg/kg/day and FFA 0.2 mg/kg/day had a ≥50% reduction in monthly drop seizures, compared with 10.3% of placebo. Overall, FFA therapy was well tolerated. Most frequent adverse events (at least 10%) included decreased appetite, somnolence, fatigue, vomiting, diarrhea and pyrexia. No cardiovascular complications were reported (Knupp 2021a). After completion of the randomized-controlled phase, patients were enrolled in the open-label extension study and received FFA twice daily for up to 1 year. After 10–12 months of treatment, among 170 patients 51.2% achieved a ≥50% reduction in drop seizures (Knupp 2021b). No patients developed valvular heart disease or pulmonary arterial hypertension. In a phase II, open-label study (NCT02655198), 13 LGS patients were administered adjunctive FFA at an initial dose of 0.2 mg/kg/day gradually increased up to 0.8 mg/kg/day in non-responders. In the 20-weeks core study 62% of patients achieved a ≥50% reduction in convulsive seizures frequency, while at 15 months 67% had a ≥50% reduction. In this patients, the most common adverse event was decreased appetite. No patient developed cardiac complications (Lagae et al., 2018).

### Fenfluramine in Others Drug-Resistant Epileptic Syndromes

Recent clinical trials conducted in small groups of patients have demonstrated the efficacy of FFA in other drug-resistant epilepsies (Table1), including CDKL5 deficiency disorder (CDD). CDD is an X-linked pharmacoresistant disorder characterized by early onset refractory epilepsy, generalized hypotonia, intellectual disability and cortical vision impairment (Jakimiec et al., 2020). In a clinical trial, 6 children with CDD were treated with FFA at 0.4 mg/kg/day or 0.7 mg/kg/day. Five patients with generalized tonic-clonic seizures at baseline achieved a ≥75% of seizure reduction after FFA treatment. Two patients with tonic seizures at baseline achieved a ≥50% of seizure reduction after FFA treatment, while the only patient with myoclonic seizures had a 71.4% of seizure reduction after FFA treatment. Adverse events including decreased appetite were reported in 2 patients, but no one developed valvular heart disease or pulmonary arterial hypertension (Devinsky et al., 2021). A phase II clinical trial of fenfluramine in patients with refractory infantile spasms is currently enrolling patients (NCT04289467). Inclusion criteria provides diagnosis of infantile spasms not responsive to adequate treatment with ACTH and vigabatrin. Enrolled patients are treated with FFA 0.8 mg/kg/day, for an initial duration of 21 days. Patients with favorable response will have an option to continue treatment for up to 6 months (ClinicalTrial.gov 2020). FFA has also been tested on a small group of patients suffering from Sunflower syndrome a rare photosensitive epilepsy. Patients with Sunflower Syndrome have the tendency to seek light sources and present highly stereotyped behaviors defined as hand waving episodes (HWE) (Belcastro et al., 2021). In this open-label study, 10 patients with Sunflower syndrome were treated with FFA at an initial dose of 0.2 mg/kg/day, subsequently increased to a maximum of 0.7 mg/kg/day. Of the 9 patients who completed the 3 months core-study, 6 achieved a ≥70% reduction in seizure frequency. No cardiac complications were observed in any of the treated patients during the observation period. The most common adverse event were fatigue, loss of appetite, rhinorrhea and diarrhea (Geenen et al., 2021).

## Conclusion

Despite many years of research, the treatment of DREs still represents a major challenges for clinicians and, of course, for patients and their families. Particularly in pediatric age, the greater impact of ethical issues and adverse effects, makes this condition even more challenging. Hence the need for new drugs that can lead to improvements in the field of pediatric epilepsies. Recently, fenfluramine has been the focus of several studies which evaluated its efficacy and safety for the treatment of DS, LGS and other refractory epilepsies. The pharmacology of fenfluramine is complex and multiple mechanisms involving both serotonergic and sigma-1 activity may work collectively to promote antiseizure activity. Both in randomized controlled trials and open-label studies, fenfluramine has proven to be effective as adjunctive therapy in reducing convulsive seizures associated with DS and to a lesser extent in LGS. This could be attributed to the more heterogeneous pathogenesis of LGS compared to DS. Echocardiographic monitoring is recommended when initiating FFA therapy. However, it is yet to clarify whether the adverse cardiovascular effects observed in adult treated with high doses of FFA (>60 mg/day) can actually translate into a pediatric population treated with lower doses. FFA showed an overall favorable profile of safety and tolerability, with mostly mild side effects, suggesting that benefits might outweigh potential cardiac risks, although this will need to be established in targeted investigations.
