# Corrigendum: Improving therapy of pharmacoresistant epilepsies: The role of fenfluramine

**DOI:** 10.3389/fphar.2022.1116575

**Published:** 2023-01-05

**Authors:** Gianluca Dini, Eleonora Tulli, Giovanni Battista Dell’Isola, Elisabetta Mencaroni, Giuseppe Di Cara, Pasquale Striano, Alberto Verrotti

**Affiliations:** ^1^ Department of Pediatrics, University of Perugia, Perugia, Italy; ^2^ Pediatric Neurology and Muscular Diseases Unit, IRCCS “G. Gaslini” Institute, Genoa, Italy; ^3^ Department of Neurosciences, Rehabilitation, Ophthalmology, Genetics, Maternal and Child Health, University of Genoa, Genoa, Italy

**Keywords:** fenfluramine, pharmacoresistant epilepsy, Dravet syndrome, Lennox-Gastaut syndrome, anti-seizure medication (ASM)

In the published article, there was an error. The conclusions of the article should be more cautionary and provide a more objective stance on the benefits and risks of this drug and its use. As such, a correction has been made to the **Conclusion**, final sentence. The original sentence reads as follows: “FFA showed an overall favorable profile of safety and tolerability, with mostly mild side effects, pointing out that significant benefits outweigh potential cardiac risks.”

The corrected sentence appears below: “FFA showed an overall favorable profile of safety and tolerability, with mostly mild side effects, suggesting that benefits might outweigh potential cardiac risks, although this will need to be established in targeted investigations.”

The authors apologize for this error and state that this does not change the scientific conclusions of the article in any way. The original article has been updated.

